# Monte Carlo simulations in radiotherapy dosimetry

**DOI:** 10.1186/s13014-018-1065-3

**Published:** 2018-06-27

**Authors:** Pedro Andreo

**Affiliations:** Department of Medical Radiation Physics and Nuclear Medicine, Karolinska University Hospital, and Department of Oncology-Pathology, Karolinska Institutet, Stockholm, SE-171 76 Sweden

**Keywords:** Monte Carlo, Radiotherapy physics, Radiotherapy dosimetry, Monte Carlo treatment planning

## Abstract

**Background:**

The use of the Monte Carlo (MC) method in radiotherapy dosimetry has increased almost exponentially in the last decades. Its widespread use in the field has converted this computer simulation technique in a common tool for reference and treatment planning dosimetry calculations.

**Methods:**

This work reviews the different MC calculations made on dosimetric quantities, like stopping-power ratios and perturbation correction factors required for reference ionization chamber dosimetry, as well as the fully realistic MC simulations currently available on clinical accelerators, detectors and patient treatment planning.

**Conclusions:**

Issues are raised that include the necessity for consistency in the data throughout the entire dosimetry chain in reference dosimetry, and how Bragg-Gray theory breaks down for small photon fields. Both aspects are less critical for MC treatment planning applications, but there are important constraints like tissue characterization and its patient-to-patient variability, which together with the conversion between dose-to-water and dose-to-tissue, are analysed in detail. Although these constraints are common to all methods and algorithms used in different types of treatment planning systems, they make uncertainties involved in MC treatment planning to still remain “uncertain”.

## Introduction

The use of the Monte Carlo (MC) method to solve problems in the field of radiotherapy dosimetry has increased almost exponentially since the 1970s [1–3]. The range of MC applications spans from the calculation of fundamental dosimetric quantities to simulations of radiotherapy treatment planning. Although computer power requirements restricted early applications to simple geometries, like infinite parallel slabs or cylinders, today’s availability of computing power allows the simulation of detailed 3-D geometries like those used for clinical accelerator treatment heads, ionization chambers and other detectors, and patient treatments using CT data. In all cases the complete phase-space that characterizes the energy, position and direction of the particles reaching a detector or a given organ within a patient, including all particle generations, can be determined. Hence, absorbed dose and other dosimetric quantities like fluence, kerma, etc can be calculated directly or performing subsequent analytical calculations.

This paper describes important MC applications in the different areas of radiotherapy dosimetry. It is initiated with a background on basic dosimetric expressions and key quantities, followed by fluence-based calculations like stopping-power ratios and mass energy-absorption ratios. Presented next is the influence of a detector placed within a homogeneous medium, causing deviations from Bragg-Gray theory, which is followed by a description of calculations on perturbation correction factors. The so-far generally accepted approach to deal with perturbation effects has raised issues in relation with the dosimetry of small megavoltage photon beams; they have led to a widely accepted alternative that avoids computing perturbation factors, an issue also discussed. The general aspects and developments on MC in radiotherapy treatment planning are summarized, followed by a short discussion on the controversy between dose-to-water and dose-to-tissue and the conversion between these two quantities. Finally, some conclusions are drawn.

## Background

### Basic dosimetry expressions

As is well-known, dosimetry is based on cavity theory (c.f., ref. [3] and references therein). Its goal is to determine the conversion factor between the absorbed dose in two media, in particular between the dose ‘measured’^1^ in a detector and that in a homogeneous medium where the dose is to be determined for a given radiation beam quality *Q*. The conversion is usually denoted by a factor *f*, defined as

1 $$ f_{\text{med,det}}(Q) = \left(\frac{D_{\text{med}}(P)}{\bar D_{\text{det}}} \right)_{Q}   $$

where *D*_med_(*P*) corresponds to the dose at a point of interest in the homogeneous medium and $\bar D_{\text {det}}$ is the mean absorbed dose within the detector. In a MC calculation it is relatively straightforward to calculate $\bar D_{\text {det}}$ as the average energy deposited by all charged particles within a volume divided by its mass, but calculating the dose at a point, *D*_med_(*P*), requires considering an infinitesimally small volume, an approach that relies on some kind of interpolation process.

Absorbed dose is usually derived from the energy loss along a given particle track-length segment, and is thus directly related to the dosimetric quantity particle fluence, *Φ*. The latter is calculated as the sum of particle tracks within a given volume, divided by the volume, hence with units cm ^−2^. It is common to compute the fluence differential in energy, *Φ*_*E*_, which has units cm ^−2^ MeV ^−1^. When this quantity is calculated by a MC simulation, either for charged particles or for photons, the absorbed dose in a medium can be determined as

2 $$ \begin{aligned} & D_{\text{med}} \mathop =^{\text{CPE}} \int_{0}^{E_{\max}} \left[ \Phi_{E} \right]_{\text{med}} \left[S_{\text{el}}(E)/\rho \right]_{\text{med}}\,\mathrm{d}E \,\,\,\,\,\,{\text{for charged particles}}\\ & D_{\text{med}} \mathop =^{\text{PCPE}} \int_{0}^{k_{\max}} k \, \left[ \Phi_{k} \right]_{\text{med}} \left[ \mu_{\text{en}}(k)/\rho \right]_{\text{med}}\,\mathrm{d}k \,\,\,\,\,\,{\text{for photons}} \end{aligned}   $$

where *S*_el_(*E*)/*ρ* is the mass electronic stopping power (formerly called collision stopping power, see ICRU Report 85 [4]) at the charged-particle energy *E*, *μ*_en_(*k*)/*ρ* is the mass energy-absorption coefficient of the material at the photon energy *k*, and *Φ*_*E*_ and *Φ*_*k*_ are the charged-particle and photon fluence differential in energy in the medium, respectively. Identical expressions can be formulated for the absorbed dose within a detector replacing “med” by “det”. The acronyms over the equal signs refer to the type of charged-particle equilibrium, either that of all type of charged particles generated in the medium (CPE), or to “partial charged-particle equilibrium” (PCPE) (often referred to as transient charged-particle equilibrium, TCPE). These are important remarks, because if there is no CPE the quantity determined is not absorbed dose, but *cema*, *C*, or *restricted cema*, *C*_*Δ*_, and if there is not PCPE the quantity approximates *kerma* (note that strictly *K* involves $\mu _{\text {tr}}(k) = \mu _{\text {en}}(k)/(1 - \bar g)$, $\bar g$ being the mean fraction of the kinetic energy of liberated charged particles lost in radiative processes, which depending on the photon energy might be non-negligible).

**Fig. 1:**
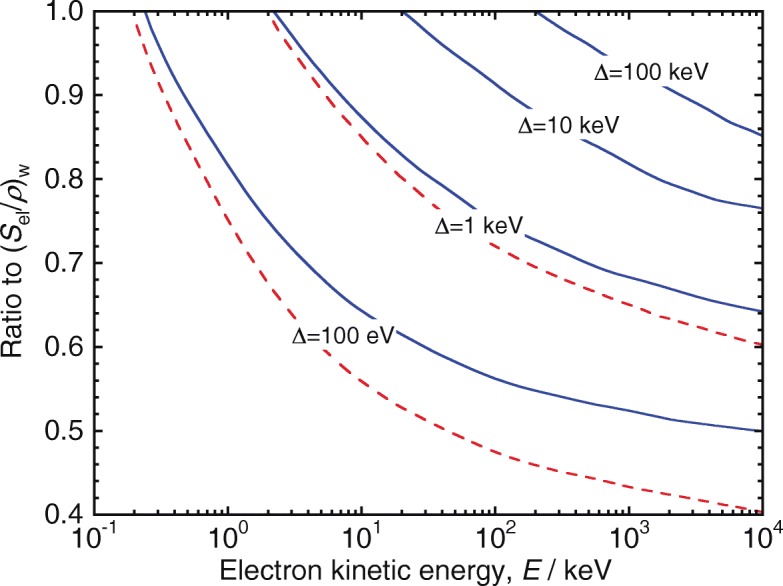
Ratios to the unrestricted stopping power in water, *S*_el_(*E*)_w_, of the LET *L*_*Δ*_(*E*) (solid lines), and of the restricted stopping power *S*_el_(*E*,*Δ*) (dashed lines), for different values of *Δ*, as a function of the incident electron kinetic energy. Adapted from ref. [3] using data from ref. [60]
(ii) ***The cavity is large compared to the electron ranges involved.***
  In this case *f* can be identified with the ratio of mass energy-absorption coefficients averaged over the photon spectrum, and charged-particle tracks are assumed to be inside the cavity. Assuming again that the cavity does not perturb the photon fluence, i.e., (*Φ*_*k*_)_med_≈(*Φ*_*k*_)_det_, the mass energy-absorption coefficients ratio, [*μ*_en_(*k*)/*ρ*]_med,det_, is defined as:
  8 $$ \begin{aligned}f_{\text{med,det}}(Q) \equiv \left[\mu_{\text{en}}/\rho \right]_{\text{med,det}} =\frac {\int\limits_{0}^{k_{\max}} k \, \left[\Phi_{k} \right]_{\text{med}}\, \left[\mu_{\text{en}}(k)/\rho \right]_{\text{med}}\,\mathrm{d}k} {\int\limits_{0}^{k_{\max}} k \, \left[\Phi_{k} \right]_{\text{med}}\, \left[\mu_{\text{en}}(k)/\rho \right]_{\text{det }}\,\mathrm{d}k} \end{aligned}  $$
  where the different quantities have been defined above. Note that [*μ*_en_(*k*)/*ρ*]_med,det_ is a quotient of energy fluence-weighted averaged mass energy-absorption coefficients.
  Recall that the mass energy-absorption coefficient, *μ*_en_/*ρ*, accounts for the local energy deposition by photon-generated charged particles, i.e., it excludes the radiative energy that escapes the local volume. The latter is, on the other hand, included in the mass energy-transfer coefficient, *μ*_tr_/*ρ*, which accounts for the photon energy transferred to kinetic energy of the generated charged particles (see, e.g., ref. [3]).

To determine the cavity-theory conversion factor *f*, which in what follows will always be averaged over the relevant charged-particle or photon spectrum, there are basically two different cases:

(i) ***The cavity is small compared to the charged-particle ranges involved.***
  In this case *f* is identified with the stopping-power ratio, and charged-particle tracks are assumed to cross the cavity. Assuming also that the cavity does not perturb the primary charged-particle fluence $\Phi _{E}^{\text {prim}}$, i.e., that it conforms the fundamental Bragg-Gray approximation *Φ*_med_≈*Φ*_det_, the so-called Bragg-Gray stopping-power ratio is defined as:
  3 $$ {}f_{\text{med,det}}(Q) \equiv s_{\text{med,det}}^{\text{BG}} =\frac {\int\limits_{0}^{E_{\max}} \left[ \Phi_{E}^{\text{prim}}\right]_{\text{med}} \left[ S_{\text{el}}(E)/\rho \right]_{\text{med}} \, \mathrm{d}E } {\int\limits_{0}^{E_{\max}} \left[ \Phi_{E}^{\text{prim}}\right]_{\text{med}} \left[ S_{\text{el}}(E)/\rho \right]_{\text{det}} \, \mathrm{d}E }   $$
  which is a quotient of fluence-weighted average mass stopping powers resulting in a ratio of absorbed doses (or of cemas if there is no CPE). Note that whereas the fluence is the same in the numerator and denominator, mass stopping powers correspond to each material, “med” and “det”, respectively. It is also emphasized that the primary charged-particle fluence does not include knock-on electrons (delta rays) or the secondary and higher-order charged particles created by the primary particles.
  A refinement in the theory is made for Spencer-Attix stopping-power ratios, where the charged-particle fluence, differential in energy, includes knock-on electrons and any other generated charged particles having energies higher than a given threshold energy, *Δ*, related to the cavity size (its mean chord length), in addition to the primary particles. In this case the fundamental Bragg-Gray approximation is $\left (\Phi _{E}^{\text {tot}}\right)_{\text {med}} \approx \left (\Phi _{E}^{\text {tot}}\right)_{\text {det}}$. Additionally, a so called *track-end* term is added that accounts for the energy deposited by electrons with energies below *Δ*. The Spencer-Attix stopping-power ratio is defined as:
  4 $$ \begin{aligned} &f_{\text{med,det}}(Q) \equiv s_{\text{med,det}}^{\text{SA}} =\\ & \frac {\int\limits_{\Delta}^{E_{\max}} \left[\Phi_{E}^{\text{tot}}\right]_{\text{med}} \, \left[S_{\text{el}}(E,\Delta)/\rho\right]_{\text{med}}\, \mathrm{d}E \,+\, \left[\Phi_{E}^{\text{tot}}(\Delta)\right]_{\text{med}}\, \left[S_{\text{el}}(\Delta)/\rho\right]_{\text{med}} \, \Delta } {\int\limits_{\Delta}^{E_{\max}} \left[\Phi_{E}^{\text{tot}}\right]_{\text{med}} \, \left[S_{\text{el}}(E,\Delta)/\rho\right]_{\text{det}}\, \mathrm{d}E \,+\, \left[\Phi_{E}^{\text{tot}}(\Delta)\right]_{\text{med}}\, \left[S_{\text{el}}(\Delta)/\rho\right]_{\text{det}} \, \Delta }  \end{aligned}  $$
  which is a quotient of fluence-weighted average restricted mass stopping powers resulting in a ratio of absorbed doses (or restricted cemas, *C*_*Δ*_ [5] 2, if there is no CPE), and the track-end terms include the fluence and the relevant (unrestricted) stopping power at the threshold energy *Δ*. Note that as the fluence includes now all kind of charged-particles, is termed $\Phi _{E}^{\text {tot}}$.
  It should be emphasized that, according to the definition of *linear energy transfer* (LET) given in ICRU Report 85 [4], the restricted stopping power is not identical to the LET, *L*_*Δ*_, for very low values of the energy threshold or cut-off *Δ*, as is often formulated.
  It is of interest to recall that the restricted electronic stopping power *S*_el_(*E*,*Δ*) provides the component of a charged-particle kinetic energy lost in inelastic collisions with atomic electrons that is deposited “locally”. This corresponds to a volume whose dimensions are limited by the range of ejected secondary electrons having an energy *Δ*. The energy loss $\mathcal W$ is then restricted to a maximum value *Δ*. Hence, while the unrestricted stopping power, *S*_el_(*E*), considers the sum of all energy losses $\mathcal W$ up to a maximum value ${\mathcal {W}}_{\text {max}}$ (*E*/2 for electrons and *E* for positrons), *S*_el_(*E*,*Δ*) excludes energy losses in the interval $\Delta < {\mathcal {W}} \le {\mathcal {W}}_{\text {max}}$. A secondary electron ejected from the atomic *i*-shell has a kinetic energy $\varepsilon _{i}={\mathcal {W}} -U_{i}$, where *U*_*i*_ is the electron binding energy of the shell. Note then that *S*_el_(*E*) includes the sum of all the kinetic energies of secondary electrons, *ε*_ke_, plus their binding energies *U*_B_, i.e.,
  5 $$ S_{\text{el}}(E)=\varepsilon_{\text{ke}}+U_{\mathrm{B}},  $$
  whereas *S*_el_(*E*,*Δ*) includes the sum of secondary-electron kinetic energies below *Δ*, *ε*_ke≤*Δ*_, plus their binding energies, i.e.
  6 $$ S_{\text{el}}(E,\Delta)=\varepsilon_{\text{ke} \le \Delta}+U_{\mathrm{B}},  $$
  The new definition of LET, *L*_*Δ*_(*E*), given by ICRU-85 excludes from *S*_el_(*E*) the kinetic energy of secondary electrons when is higher than *Δ*; it does not exclude their binding energy. Hence,
  7 $$ L_{\Delta} (E) = S_{\text{el}}(E) - \varepsilon_{\text{ke} > \Delta},  $$
  where *ε*_ke>*Δ*_ is the sum of the kinetic energies of secondary electrons above *Δ*. As a consequence, *L*_*Δ*_(*E*)=*S*_el_(*E*) for *E*≤*Δ*, and for low energies *L*_*Δ*_(*E*)>*S*_el_(*E*,*Δ*). Reference [3] could be consulted for further details. Figure 1 shows ratios *L*_*Δ*_(*E*)/*S*_el_(*E*) and *S*_el_(*E*,*Δ*)/*S*_el_(*E*) for different values of *Δ*, as a function of the incident electron kinetic energy, where significant discrepancies between the two ratios can be observed for values of *Δ* below 10 keV.

A third option exists for intermediate cases, based on Burlin’s cavity theory, which combines the approaches (i) and (ii) described above, but, from a MC calculation point of view, there is no difference in the way in which *s*_med,det_ and [*μ*_en_/*ρ*]_med,det_ are evaluated.

### Dosimetric key quantities

From the previous section one can infer that the key quantities needed for cavity theory are mass stopping powers, restricted *S*_el_(*E*,*Δ*)/*ρ* and unrestricted *S*_el_(*E*)/*ρ*, for different types of charged-particles, and mass energy-absorption coefficients *μ*_en_(*k*)/*ρ*, although Monte Carlo calculations involve also mass radiative stopping power *S*_rad_(*E*)/*ρ*. Both *S*_el_(*E*)/*ρ* and *S*_el_(*E*,*Δ*)/*ρ* have an appreciable dependence with the fundamental quantity *mean excitation energy*, the so called *I*-value. Interested readers can find a detailed description of the formulation of the mean excitation energy in ICRU Report 90 [5]; the impact of the *I*-value in radiotherapy dosimetry has been discussed at length in refs. [3, 6, 7].

Involved also in certain dosimetric expressions, like in the beam quality factor $k_{Q,Q_{0}}$ for reference dosimetry (e.g., in the Code of Practice IAEA TRS-398 [8]), is the *mean energy to create an ion pair in air*, the *W*_air_-value, which for high-energy electrons and photons has the value *W*_air_=33.97 eV or *W*_air_/*e*=33.97 J C ^−1^, *e* being the elementary charge.

All the quantities above have recently been updated for water, air and graphite by ICRU Report 90 [5], superseding the values given in ICRU Reports 37 [9] and 49 [10], and now being adopted by standard laboratories. As a consequence, the basic data used in MC simulations, and for most of the available stopping-power ratios *s*_med,det_ and ratios of mass energy-absorption coefficients [*μ*_en_(*k*)/*ρ*]_med,det_, should be updated to avoid breaking the consistency of the dosimetry chain. (Note that there is an on-going IAEA project to update TRS-398 on this regard).

## Monte Carlo calculation of dosimetric quantities

### Stopping-power ratios for reference dosimetry

Once the dosimetric key quantities have been adopted, both in the MC code at hand and for solving the cavity integrals, the problem basically is restricted to the calculation of the relevant charged particle (primary or total) or photon fluence, differential in energy, by scoring *track-length spectra*.

It is not an overstatement to claim that many of the MC advances in the field have been related to developments on electron transport algorithms, especially in the presence of interface boundaries, where track-length segments may become so short that multiple scattering theories are no longer valid under the so-called *condensed history technique* developed by Berger more than 50 years ago [11]. Many of the current MC systems include such technique, rather than the interaction-by-interaction (single scattering) type of simulation often used for low-energy transport simulation. An extreme case to deal with is that of an air-filled ionization chamber, whose detailed simulation has posed a considerable challenge for years, to the extreme that only two of the MC systems generally available, *PENELOPE* [12] and *EGSnrc* [13], can yield accurate results^3^. Computer codes based on these systems can switch from multiple- to single-scattering physical models whenever track-length segments shorten at the proximity of an interface boundary. Except at low photon energies, where photoabsorption and atomic radiative and non-radiative transitions need to be accounted for properly, photons are in principle simulated in a rather straightforward manner, interaction-by-interaction. However, the large number of generated secondary and higher-order electrons brings us to the beginning of this paragraph: there is no reliable photon transport simulation without an accurate treatment of electron transport.

The calculation of dosimetric quantities and correction factors for radiotherapy measurements can be considered to have been initiated by the work of Berger and Seltzer [14], whose results served to benchmark other MC codes in the early days. As is well-known, a basic dosimetric quantity for absorbed dose determination using ionization chambers is the water-to-air stopping-power ratio, *s*_w,air_, which is determined from MC-calculated electron fluence using Eqs. (3) and (4). This type of calculations was pioneered by Berger et al. [15] for electron beams using slowing-down spectra at different depths; they were later improved by Nahum [16] for the evaluation of Spencer-Attix *s*_w,air_ values including the track-end term. For photon beams, Nahum [16] calculated for the first time stopping-power ratios, scoring electron fluence spectra at various depths and solving subsequently the relevant cavity integrals introducing the track-end term. Values of *s*_w,air_ correlated with clinical photon beam quality specifiers were computed by multiple authors (see, e.g., refs. [17–20]), as well as for electron beams [21], providing the data included in most dosimetry protocols like IAEA TRS-398 [8] and AAPM TG-51 [22]. It should be pointed that most of the currently available *s*_w,air_ data performs the calculations internally during the MC simulation, rather than computing *Φ*_*E*_ first and evaluating subsequently the cavity integrals described above.

**Fig. 2:**
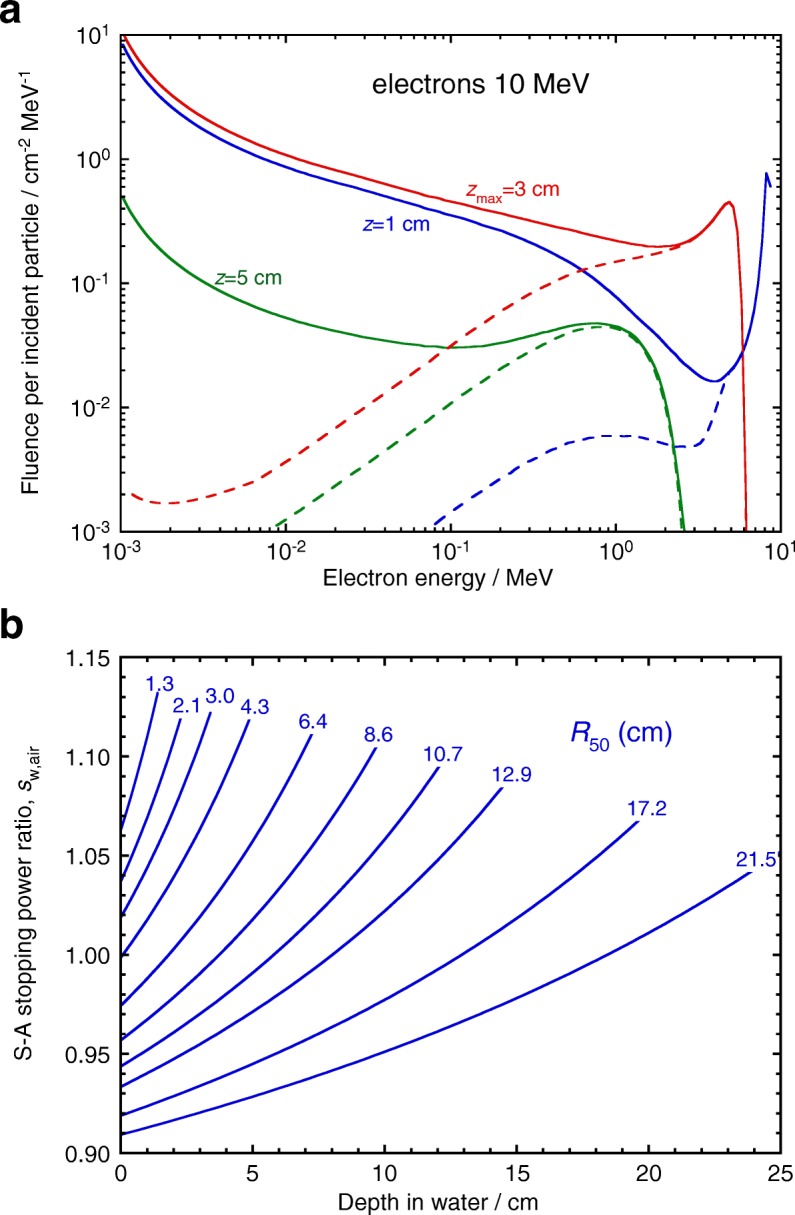
**a** Monte Carlo calculated electron fluence spectra for a 10 MeV broad electron beam at three depths (solid lines: total spectra; dashed lines: primary electrons). **b** Depth variation of Spencer-Attix (*Δ*=10 keV) stopping-power ratios, water-to-air, for clinical electron beams as a function of *R*_50_. Note the large variation of the electron spectra with depth, resulting in the strong depth dependence of the *s*_w,air_ values. Adapted from ref. [3]

Figure 2a shows MC-calculated electron fluence spectra for a 10 MeV electron beam, where primary and total electron spectra are plotted at three depths. The total electron spectra at a large number of depths is used to compute *s*_w,air_-values using Eq. (4) for different clinical beams, of quality expressed by their *half-value depth*, *R*_50_, see Fig. 2b. The large variation of the electron spectra with depth should be emphasized, which results in the strong depth dependence of the *s*_w,air_ values.

**Fig. 3:**
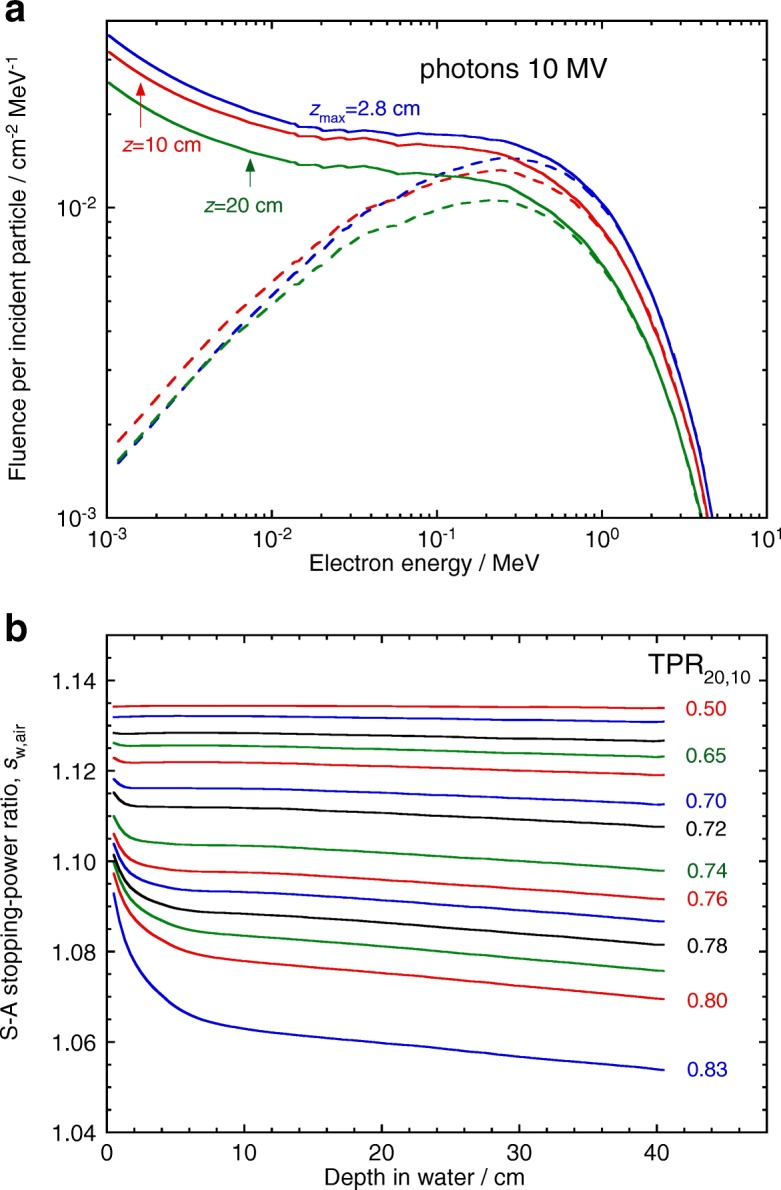
**a** Monte Carlo calculated electron fluence spectra for a 10 MV broad photon beam at three depths (solid lines: total spectra; dashed lines: primary electrons). **b** Depth variation of Spencer-Attix (*Δ*=10 keV) stopping-power ratios, water-to-air, for clinical photon beams as a function of TPR _20,10_. Note the almost constant depth dependence of the electron spectra, resulting in practically depth-independent *s*_w,air_ values. Adapted from ref. [3]

The corresponding case for photons is illustrated in Fig. 3, where primary and total electron spectra generated by a 10 MV photon beam are plotted at three depths, see Fig. 3a. Stopping-power ratios are calculated with Eq. (4) for different clinical beams, of quality expressed by the *tissue-phantom ratio* at 10 and 20 cm depth, TPR _20,10_, in Fig. 3b. In this case, the small depth dependence of the electron spectra results in practically depth-independent *s*_w,air_ values except at the highest energies.

### Influence of the detector: perturbation factors

As emphasized for Eqs. (3) and (4), the calculation of stopping-power ratios is based on the fundamental Bragg-Gray CPE approximation *Φ*_med_≈*Φ*_det_ for the electron spectra. Inserting a detector in the medium results in a change in the electron spectrum within the detector radiation sensitive volume relative to that in the homogeneous medium, i.e., CPE strictly fails due to the influence of the detector size, shape and construction materials. The effect is known as a *perturbation*.

Classically, the departure from Bragg-Gray conditions has been dealt with introducing a so-called detector *perturbation correction factor* and assuming that the approximation *Φ*_med_≈*Φ*_det_ is still valid for stopping-power ratios. Hence, the corrected expression for *f*_med,det_(*Q*) becomes

9 $$ f_{\text{med,det}}(Q) = s_{\text{med,det}}(Q) \; p_{\text{det}}(Q)   $$

which leads to

10 $$ D_{\text{med}} = \bar D_{\text{det}} \; s_{\text{med,det}}(Q) \; p_{\text{det}}(Q)   $$

The major advantage of this approach is that one can still rely on conventional stopping-power ratios based on the assumption of unperturbed electron fluence. There is, however, a major constraint imposed by the conditions for the validity of the approximation, as *perturbation correction factors must be small*, and be then assumed to be *independent of each other*. Under these conditions, various types of perturbation factors have been proposed to describe the influence of the different detector components or effects, by writing

11 $$ p_{\text{det}}(Q) = \prod\limits_{i} p_{\text{det,i}} = p_{\text{dis}}\, p_{\text{wall}}\, p_{\text{fl}}\, p_{\text{cel}}\, p_{\text{stem}} \ldots   $$

where *p*_dis_ accounts for the effect of replacing a volume of water by that of the detector, *p*_wall_ accounts for the presence of non-water-equivalent materials in the detector body and walls, *p*_fl_ corrects for the intrinsic difference in fluence between water and the detector volumes, and *p*_cel_ and *p*_stem_ correct for the presence of a central electrode and stem, respectively, if they are relevant to the type of detector involved.

The MC calculation of perturbation correction factors for ionization chambers and other types of detectors has received special consideration due to the electron simulation difficulties mentioned in the previous section. The first MC calculations on ionization chamber correction factors were made for ^60^Co in-air measurements, and the simulations by Bond et al. [23], Nath and Schulz [24] and McEwan and Smyth [25] deserve being mentioned. Their results showing a dependence with the chamber dimensions contradicted, however, Bragg-Gray theory and prompted critical publications by others that revealed the importance of interface effects (mostly related to multiple scattering). These led to the development of an algorithm termed *EGS4/PRESTA* [26] that made simulations of chambers more accurate, obtaining uncertainties of the order of 1%, a very good figure in the late 1980s. The approach was improved later on by a *PRESTA2* algorithm, which is included in the *EGSnrc* MC system). The *PENELOPE* system, on the other hand, uses a different approach and has never been affected by the kind of interfaces effects shown by the *EGS4* system.

At the time when the interface effects were realized, Smyth [27] demonstrated that the conditions required by Fano’s theorem for CPE conditions in a medium could be simulated with a fictitious experiment. This considered a cavity filled with the same material as the surrounding medium, but in a gas-like form, i.e., having the same cross sections but a very large difference in mass density. Simulations of this experiment were made by Seuntjens et al. [28] using *EGSnrc* and by Sempau and Andreo [29] using *PENELOPE*. The agreement with Fano’s theorem was of the order of 0.1%, a level that no other MC system has been able to achieve so far. The state-of-the-art for this type of calculations is that linacs phase-space data (see next section) are used as radiation sources to simulate the response of ionization chambers based on the detailed description of their geometry.

**Fig. 4:**
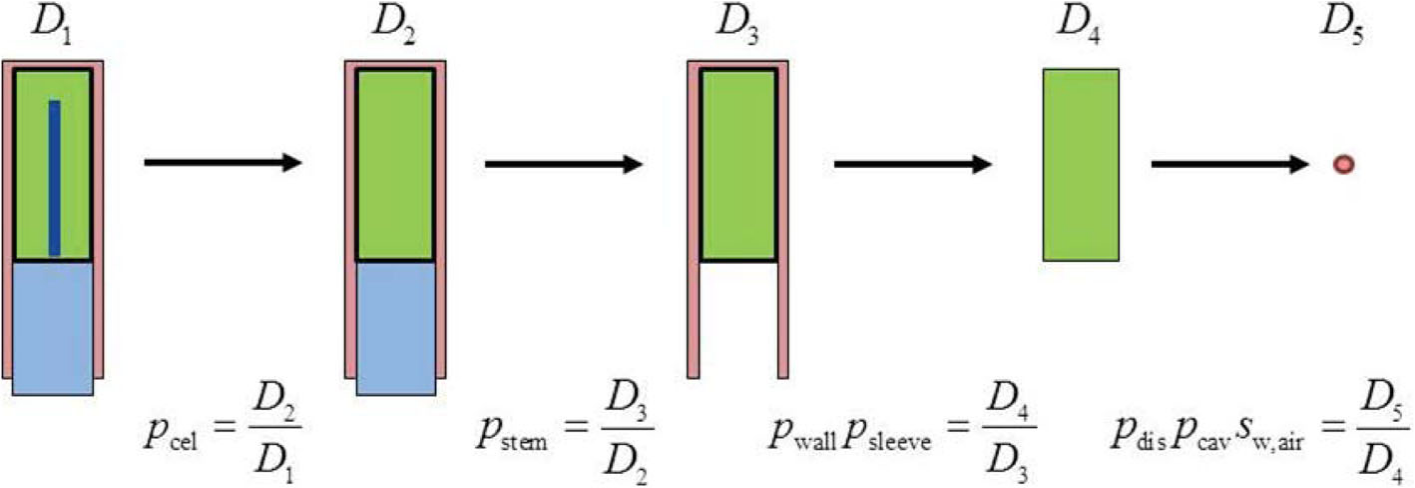
Chain of dose ratios to calculate ionization chamber perturbation factors. Dose ratios in the chamber cavity are defined in steps that include different chamber components (*D*_1_−*D*_4_). The final step includes the dose to a small volume of water (*D*_5_). Adapted from ref. [30]

An interesting development for the MC calculation of perturbation correction factors has been the work of Wulff et al. [30], where a chain of dose ratios, which includes the effect of different chamber components, is used to derive the different *p*_det,*i*_ factors in Eq. (11); the technique is illustrated in Fig. 4.

**Fig. 5:**
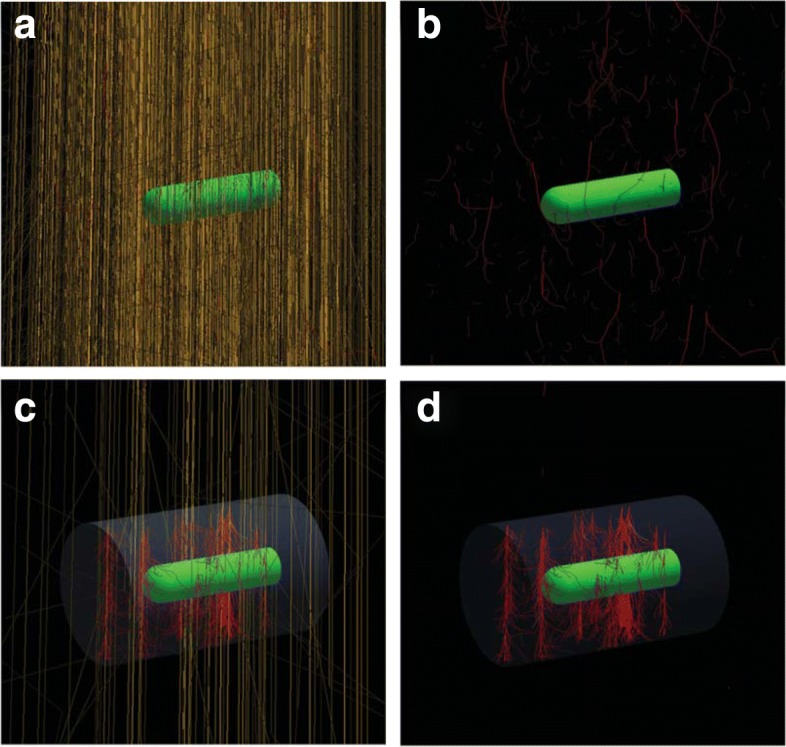
Examples of track simulations obtained with the MC code *egs_chamber* using cross-section enhancement. **a** Normal particle transport during the simulation of a ionization chamber within a phantom irradiated by 10^3^ 6 MV photons; **b** secondary electrons tracks in panel (**a**). **c** Transport of 10^2^ photons using cross-section enhancement in a volume surrounding the chamber; **d** secondary electrons tracks in panel (**c**). Courtesy from J. Wulff

It is implemented in Wulff’s *egs_chamber* user code, which includes an ample set of variance reduction techniques like, e.g., *correlated sampling* and *local photon cross-section enhancement*. The latter increases the density of electron tracks within the chamber and a surrounding volume, leading to an overall efficiency gain of up to 10^4^ that allows relatively fast calculations with type A uncertainties of the order of 0.1%. Examples of track simulations obtained with this code are illustrated in Fig. 5, where panel (a) shows tracks under normal particle transport during the simulation of a ionization chamber within a phantom irradiated by 10^3^ 6 MV photons, their extracted electron tracks being shown in panel (b); panels (c) and (d) show the dramatic increase in electron density tracks within a volume surrounding the chamber following the transport of only 10^2^ photons with cross-section enhancement in that volume.

In recent years a large number of calculations similar to those described above have been made for the dosimetry of small megavoltage photon fields, where other types of detectors have been simulated. Correction factors have been calculated using MC for mini and micro ionization chambers, silicon diodes, natural and synthetic diamonds, etc. The resulting data have been included in the IAEA TRS-483 Code of Practice for the dosimetry of small static megavoltage photon beams (c.f. ref. [31] and references therein).

### Issues on calculated perturbation factors

The applicability of the Bragg-Gray approximation commented above raises a rather special type of issue in the MC calculation of perturbation factors.

It should be stated first that an absorbed dose calculation made with MC does not require CPE. However, our current formulations for *f*_med,det_(*Q*) (e.g., stopping-power ratios) rely on CPE-based expressions. The condition for the current Bragg-Gray approach, i.e., assuming that *Φ*_med_≈*Φ*_det_ and that the different perturbation corrections *p*_det,*i*_ in Eq. (11) are independent, still requires *small* perturbation correction factors to be able to assume that they are independent of each other.

In recent years it has been realized that, in small megavoltage photon beams, the MC calculated correction factors for many specific detectors can be very large (up to ∼ 10*%* for small ionization chambers) and often CPE is lacking. This means that the Bragg-Gray assumptions used so far break down.

For MC calculations simulating ionization chambers, Sempau et al. [32] proposed computing directly within the simulation the factor

12 $$ f_{\text{ch}}(Q) = \left[ \frac{D_{\mathrm{w}}(P)}{\bar D_{\text{ch-air}}} \right]_{Q}   $$

where $\bar D_{\text {ch-air}}$ and *D*_w_(*P*) are the MC-calculated mean absorbed dose in the chamber cavity and the dose to a point in water (a very small volume), respectively. Note that for conventional field sizes (i.e., non-small beams) Eq. (12) corresponds to Eq. (9), and that no specific perturbation correction factors are explicitly included.

It can then be concluded that solving the fluence-based cavity integrals (always assumed to be under CPE), discussed in the Background section, is no longer needed for practical dosimetry. In addition, it can also be stated that the assumption of small and independent *p*_det,*i*_ should no longer be needed in dosimetry.

The procedure in Eq. (12), which can be referred to as a *global* *f*_ch_(*Q*) that includes *s*_w,air_ and all possible perturbations, irrespective of their size or interrelation (e.g., not being completely independent), has become the currently accepted MC calculation approach. It differs from that used by other authors (e.g., refs. [33, 34]), where instead of the dose to a point, *D*_w_(*P*), the dose to water was calculated in a volume identical to that of the chamber, *D*_w_(v*ol*); it should be recalled, however, that Bragg-Gray theory yields the absorbed dose at one point in the medium.

Detailed fluence spectra and subsequent perturbation-correction calculations will, however, continue to be useful for analysing the influence of different components in the design of detectors (or for pedagogic purposes). For this purpose, electron fluence inside detectors where the composition of certain components can be varied (see, e.g. ref. [35]), provides a very efficient MC tool.

## Monte Carlo treatment planning (MCTP)

Since the 1990s, a number of fruitful MC developments have been made for the direct calculation of dose distributions within a patient using linacs phase-space data impinging on 3-D CT images. There were some early developments (see, e.g., an early MC review by this author [1]), but realistic MCTP could not be implemented and become a reality as a clinical tool until today’s considerable computing power was available.

At this point it is interesting to recall that the simulation of accelerator treatment heads was pioneered by the work of Petti et al. [36], Mohan et al. [37] and Udale [38], all using the *EGS4* system [39]. Currently, the *EGSnrc*-based *BEAM* user code [40] is probably the most widely used piece of software for this purpose; it was developed within a major project called *OMEGA*, designed for treatment planning purposes [41, 42]. Other MC systems incorporating accurate geometry packages like *MCNP6* [43], *PENELOPE* [12] and *GEANT4* [44] have been used to simulate specific accelerator models, and user codes like the *GEANT4*-based *GAMOS* [45], and the *PENELOPE*-based *PENLINAC* [46], *PENEASYLINAC* [47] and *PRIMO* [48] have been developed and are in current use.

“Contemporary” developments in MCTP include the already mentioned *OMEGA* project, the *Macro MC (MMC)* code designed for electron treatment planning [49], the *VMC* and *XVMC* codes [50, 51]), the *PEREGRINE* system [52] focused on photon calculations, and the *PENELOPE*-based *DPM* [53] and *PRIMO* [48]. The latter is rather unique in the sense of being a comprehensive system that includes in a single package the simulation of linacs and patient dose-planning calculations (plus a number of beam analysing graphical tools). Some of the MC codes or systems mentioned are implemented in commercial treatment planning systems (TPS), while *DPM* and *PRIMO* are free-software packages.

**Fig. 6:**
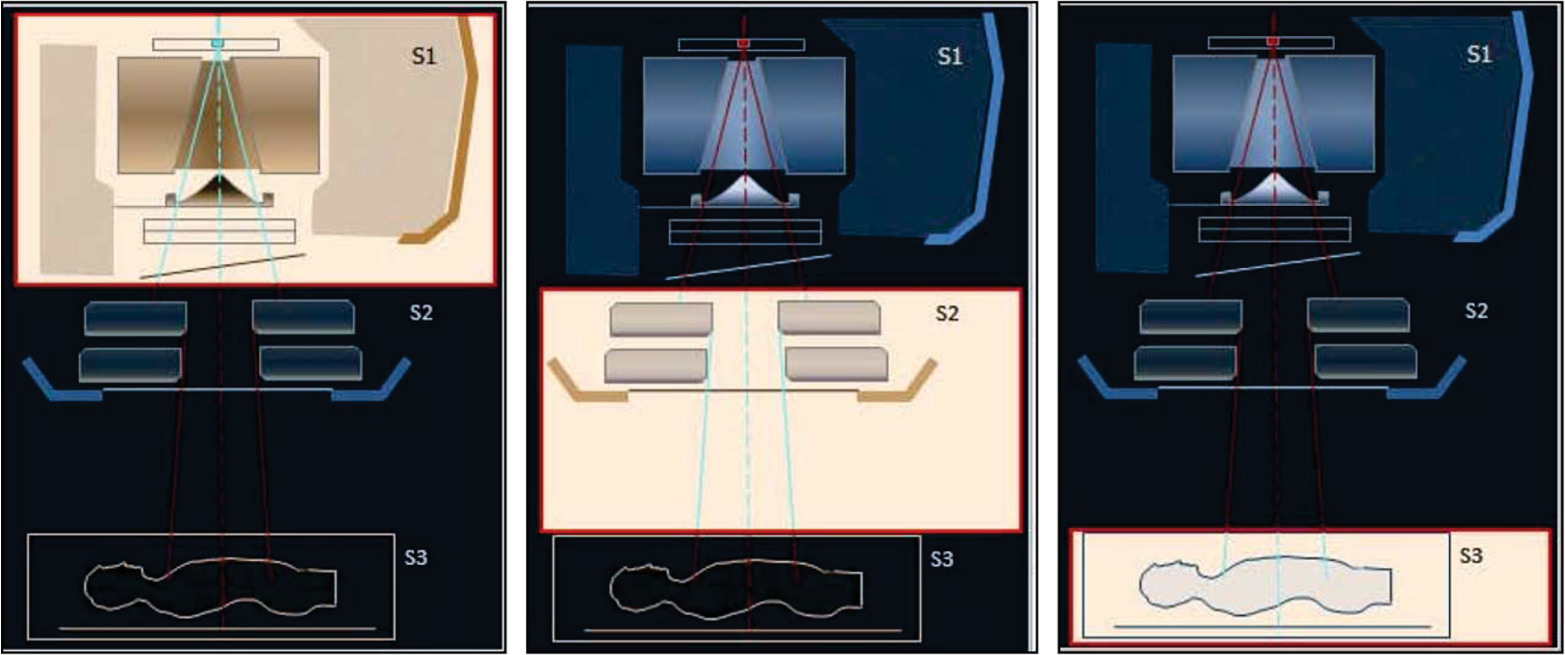
Illustration of the three general calculation steps used in Radiotherapy Monte Carlo Treatment Planning (MCTP). S1 determines phase-space data after the primary linac collimators, S2 computes phase-space data after the secondary or multileaf collimators defining the radiation field, and S3 calculates the dose distribution for the patient-specific CT geometry. Adapted from the PRIMO user’s manual [61]

Since its early development, MCTP is generally based on three calculation steps: (i) determination of the phase-space data after the primary set of linac collimators, which is a machine but not patient-specific calculation; (ii) phase-space data after the secondary or multileaf collimators, which define the radiation field for a given treatment; and (iii) simulation of the patient-specific CT geometry where the dose-planning distribution is computed. Figure 6, adapted from the PRIMO project, illustrates the three steps.

In favour of the use of MCTP it could be argued that while most analytical-based algorithms for treatment planning are adequate for calculations in homogeneous media, they have been shown to be rather crude approximations whenever inhomogeneities are present. MC simulations are not constrained a priori by the composition and/or density of the medium, and their superior dose distributions over analytical-based calculations have been thoroughly demonstrated (see the textbook [54] and references therein). Additionally, cost-free MC packages and sufficient computer power are available today at most desks. MC has then become an ideal tool for the simulation of radiation transport using any media and geometry, and MCTP is claimed to yield results within the requirements for TPSs even with inhomogeneities (experimentally verified dose differences are smaller than 2%).

Unfortunately, for sake of speed, some of the commercial MCTPs are based on *MC codes trimmed for low-Z media*, limiting for instance the number of materials they can handle (i.e., grouping similar tissues). A related issue is that of the *ALARA uncertainty* in MCTP, which will be discussed next.

### Issues on MCTP

There are three major questions that can be posed on MCTP, some of them being also applicable to all kind of TPSs (see, e.g., refs. [55, 56] and references therein):

(i) Should MCTP calculate absorbed dose-to-tissue (*D*_tis_) or dose-to-water (*D*_w_)?
(ii) Does MCTP inherently calculate *D*_tis_ accurately?
(iii) How accurate is the conversion between *D*_tis_ and *D*_w_?

On the question about calculating dose-to-tissue or dose-to-water, different arguments have been provided in the literature:

(i) In favour of using dose-to-water:
  - *D*_w_ is the basis for current clinical experience and trials, meaning that compliance with experience, mainly developed with conventional TPSs, and with established criteria for therapeutic and normal-tissue tolerance, is required.
  - The calibration of radiotherapy beams is always made in terms of the reference absorbed dose to water, which is used for any TPS dose normalization.
(ii) In favour of using dose-to-tissue:
  - *D*_tis_ is the quantity *inherently* computed *exactly* by MCTP.
  - Differences between *D*_tis_ and *D*_w_ for “water-like tissues” is *small* and *likely* to have minimal clinical impact.
  - Converting between *D*_tis_ and *D*_w_ introduces additional uncertainty in the treatment planning process, but a relation between *D*_tis_ and *D*_w_ is still necessary because of the normalization to the beam calibration reference dose to water.

The text above shows some words that have been emphasized and deserve a detailed discussion.

**Fig. 7:**
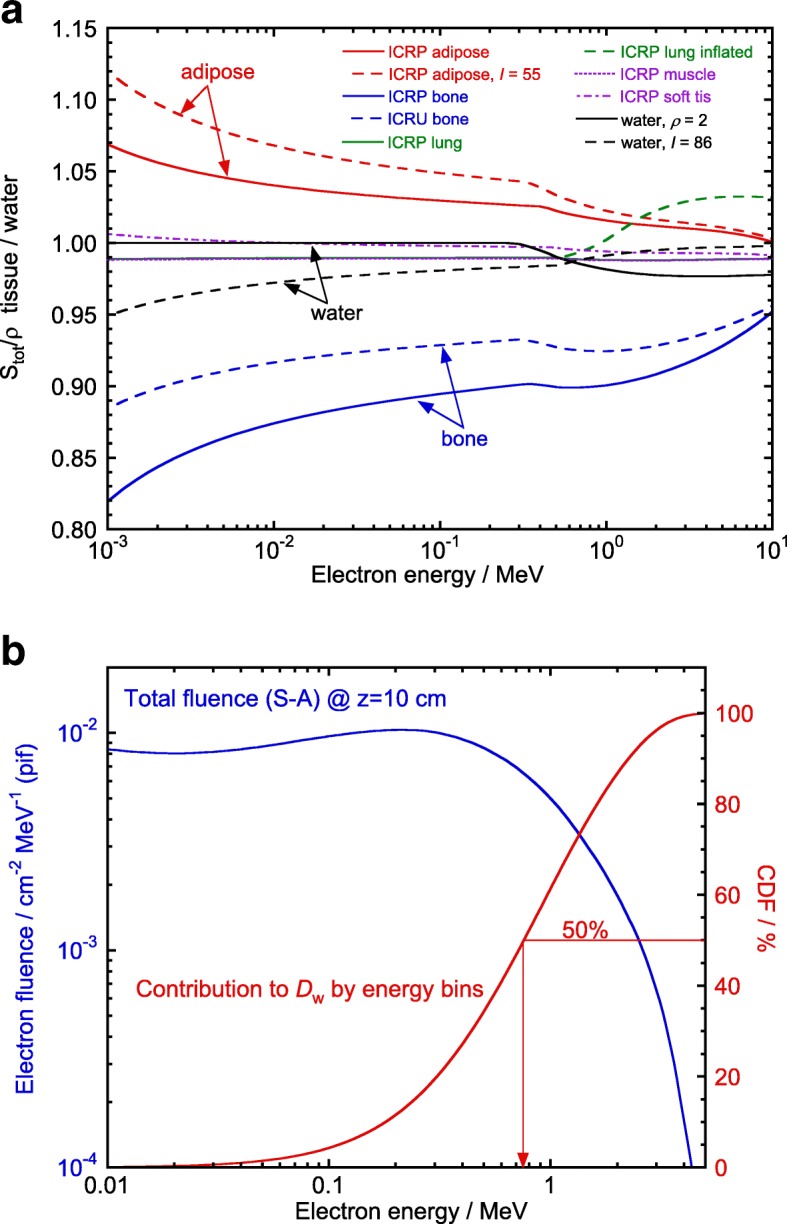
**a** Ratios of total mass stopping powers for different tissues to those for water, including values with *I*-values changed for adipose tissue, bone and water, and for *ρ*_water_=2 g cm^3^ (see text), as a function of the electron kinetic energy. **b** Monte Carlo-calculated total electron fluence differential in energy (per incident fluence) in a ∼20 cm^3^ water volume at a depth of 10 cm for 6 MV photons (left ordinate axis); the right axis corresponds to the cumulative dose fraction (CDF), showing that electrons below ∼ 0.75 MeV contribute to 50% of the absorbed dose. Adapted from ref. [55]
  The answer to the *inherently exact* character of MCTP calculations is therefore negative. Generic *I*-values represent a major limitation on any MTCP (and even on full MC systems), as individual body-tissue stopping powers are required. One can only state that MCTP has an intrinsic uncertainty (type B) which is *ALARA* (As Low As Reasonably Achievable), or rather, *ALAHBA* (As Low As the Human Body Allows). Hence, this issue questions the claimed low-uncertainty of MCTP, even if the method is still superior to that of analytical algorithms.
- The remaining important issue is the conversion between *D*_tis_ and *D*_w_, applicable to MCTP or to any other type of TPS, to relate a calculated *D*_tis_ to the reference dose to water obtained at beam calibration. The ratio between the two absorbed doses can be written as:
  13 $$ \frac{D_{\text{tis}}}{D_{\mathrm{w}}} \approx \frac {\int\limits_{0}^{E_{\max}} \Phi_{E,\text{tis}}^{\text{prim}}\; \left[S_{\text{el}}(E)/\rho\right]_{\text{tis}}\;\mathrm{d}E} {\int\limits_{0}^{E_{\max}} \Phi_{E,\mathrm{w}}^{\text{prim}}\; \left[S_{\text{el}}(E)/\rho\right]_{\mathrm{w}}\;\mathrm{d}E}   $$
  which is strictly a ratio of cemas, and only under CPE conditions the approximate sign can be replaced by equal (note that cema is used instead of restricted cema for simplicity in the formulation). This equation should not be confused with a Bragg-Gray stopping-power ratio, see Eq. (3), as it now includes the electron fluence in tissue and in water in the numerator and denominator, respectively, as fluence is different in water and in “not so water-like tissues” like bone or adipose matter. Hence, there is an implicit statement on $s_{\text {tis,w}}^{\text {BG}} \ne D_{\text {tis}}/D_{\mathrm {w}}$.
  Equation (13) points at that the widely used conversion of Siebers et al. [58], where the “converted dose-to-water” is calculated as $D_{\mathrm {w}}^{\text {conv}} = D_{\text {tis}}^{\text {MC}}\; s_{\text {w,tis}}$, does not seem to be correct. To illustrate this statement, one can be write
  14 $$ \begin{aligned} s_{\text{w,tis}} \,=\, \frac{\bar s_{\mathrm{w}}}{\bar s_{\text{tis}}} \!= &\frac{{\int\limits_{0}^{{E_{\max }}} {\Phi_{E,{\mathrm{w}}}^{{\text{prim}}}{{\left[ {{S_{{\text{el}}}}(E)/\rho} \right]}_{\mathrm{w}}}{\mathrm{d}}E} \;{\Big /}\;\int\limits_{0}^{{E_{\max }}} {\Phi_{E,\mathbf{w}}^{{\text{prim}}}{\mathrm{d}}E} }}{{\int\limits_{0}^{{E_{\max }}} {\Phi_{E{\mathrm{,tis}}}^{{\text{prim}}}{{\left[ {{S_{{\text{el}}}}(E)/\rho} \right]}_{{\text{tis}}}}{\mathrm{d}}E} \;{\Big /}\;\int\limits_{0}^{{E_{\max }}} {\Phi_{E,{\text{tis}}}^{{\text{prim}}}{\mathrm{d}}E} }}\\ \!= &\frac{{\int\limits_{0}^{{E_{\max }}} {\Phi_{E,{\mathrm{w}}}^{{\text{prim}}}{{\left[ {{S_{{\text{el}}}}(E)/\rho} \right]}_{\mathrm{w}}}{\mathrm{d}}E} }}{{\int\limits_{0}^{{E_{\max }}} {\Phi_{E,{\text{tis}}}^{{\text{prim}}}{{\left[ {{S_{{\text{el}}}}(E)/\rho} \right]}_{{\text{tis}}}}{\mathrm{d}}E} }}\;\frac{{\int\limits_{0}^{{E_{\max }}} {\Phi_{E,{\text{tis}}}^{{\text{prim}}}{\mathrm{d}}E} }}{{\int\limits_{0}^{{E_{\max }}} {\Phi_{E,{\mathrm{w}}}^{{\text{prim}}}{\mathrm{d}}E} }} \equiv \frac{{{D_{\mathrm{w}}}}}{{{D_{{\text{tis}}}}}}\;\frac{{\Phi_{{\text{tis}}}^{{\text{prim}}}}}{{\Phi_{\mathrm{w}}^{{\text{prim}}}}}  \end{aligned}  $$
  which shows that a *fluence correction factor* is required for converting between *D*_tis_ and *D*_w_, leading to
  15 $$ D_{\mathrm{w}}^{\text{conv}} = D_{\text{tis}}^{\text{MC}}\; s_{\text{w,tis}}\;\frac{\Phi_{\mathrm{w}}^{\text{prim}}}{\Phi_{\text{tis}}^{\text{prim}}}   $$
  This is a conclusion that parallels the well-known expression for reference dosimetry given in Eq. (10), where the corresponding perturbation factor can now be identified with a ratio of fluences in both media. The fluence correction factor, written as
  16 $$ k_{\Phi} = \frac{\Phi_{\mathrm{w}}}{\Phi_{\text{tis}}}   $$
  is shown in Fig. 8 for a 6 MV photon beam onto various media and tissues, including those mentioned above where their *I*-value was modified. It also includes values for “fictitious water” having densities of 2 and 10 g cm ^−3^, demonstrating that, like the mass stopping powers discussed above, fluence does not depend substantially on the density of materials provided they have identical composition.
  The magnitude of the correction factors, particularly for bone, and to a lesser extent for adipose tissues, points at the need for using a fluence correction factor, *k*_*Φ*_, if differences of up to approximately 5% are clinically relevant. Hence, the argument on differences between *D*_tis_ and *D*_w_ “being small and not having much clinical impact” is a clinical decision based on the degree of accuracy required at a given radiotherapy facility.

- With regard to the *inherently exact*characterof MCTP calculations one could argue that, in addition to the sometimes over-simplified physical models implemented in certain MCTP systems, all existing methods for tissue segmentation, where densities are obtained from CT data, and used on a look-up table to assign different tissue types, neglect patient-to-patient variation of tissue compositions, and assume that these are patient-independent (they use ICRU or ICRP compositions). This approach, used by practically all TPS types, collides with the statement by ICRU Report 44 [57]: *“It is imperative that body-tissue compositions are not given the standing of physical constants and their variability is always taken into account”*. The rationale for this categorical statement is that tissue compositions given in ICRU or ICRP reports are average values obtained from a reduced set of human-body samples, and is the main reason why stopping powers for tissues, tabulated for example in ICRU Reports 37 [9] (electrons and positrons) and 49 [10] (protons and alpha particles), are estimated to have an uncertainty of the order of 10–15%.
  To understand this uncertainty estimate, one should recall that mass electronic stopping powers, *S*_el_/*ρ*, unlike photon mass energy-absorption and energy-transfer coefficients, depend on the material density entering into the density-effect correction, *δ*, but, in addition, the full dependence of *δ* for a given medium is through $\delta _{\text {med}} = \text {function} \left [(\rho \,Z/A)_{\text {med}},I_{\text {med}}^{2},E\right ]$, which shows that *S*_el_/*ρ* depends considerably on the mean-excitation energy of the medium [7]. Obtaining this *I*-value requires the detailed atomic composition of the medium (electron distributions per shell) for a theoretical calculation, or an experimental determination using measurements with heavy-charged particles, an approach unrealistic to accomplish for individual body tissues as is done, for instance, for some compounds. Furthermore, even if tissue compositions were known, for example through MR-spectroscopy, the usual Bragg-additivity rule is a crude approximation that ignores aggregate effects, justifying the large uncertainties estimated for body-tissues stopping powers.
  Calculations reported in ref. [55] for certain tissues, where their *I*-values were changed by ± 15 *%* with respect to the nominal values given in ICRU-37, showed substantial discrepancies in the respective stopping powers; they are illustrated in Fig. 7a. The differences, shown for a change in *I*_adipose_ from 63.2 to 55 eV, in bone for *I*_bone_ values of 91.9 and 106.4 eV (as given by ICRU and ICRP, respectively, both for the same *ρ*_bone_=1.85 g cm ^−3^), and in water for *I*_w_ from 75 to 86 eV plus a fictitious case for water with *ρ*_w_=2.0 g cm ^−3^, are evident and reach up to several percent. The differences are clearly higher at low electron energies but, as is shown in Fig. 7b, for a 6 MV photon beam 50% of the dose in a water cylindrical volume of 5 cm diameter and 1 cm height at 10 cm depth is due to electrons below 0.75 MeV approximately.

**Fig. 8:**
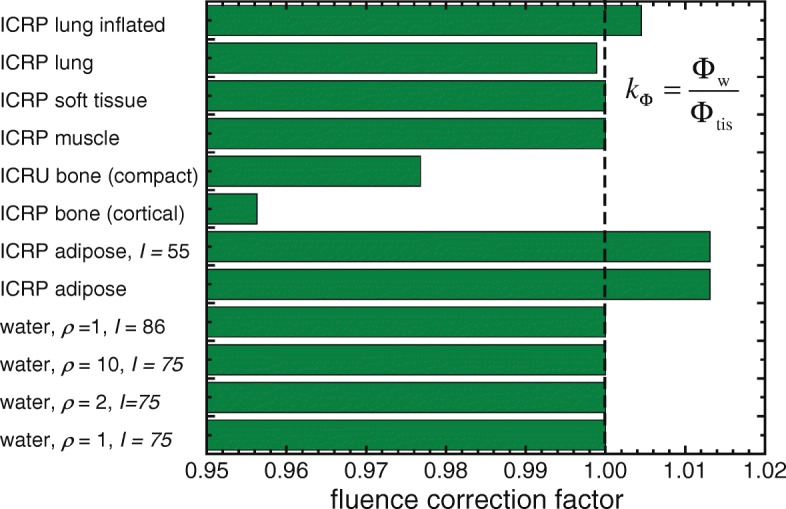
Monte Carlo-calculated fluence correction factors, *k*_*Φ*_, for the conversion between dose-to-tissue, *D*_tis_, and dose-to-water, *D*_w_, for various tissues and water media using a 6 MV photon beam. Adapted from ref. [55]
  Similar calculations to those described for high-energy photons have been done in ref. [59] for dose conversions in brachytherapy dosimetry with ^125^I, ^131^Cs and ^103^Pd sources, where the short electron ranges involved (corresponding to the case of a large detector in the Background section), make the correction factor to be in terms of an energy-fluence ratio, i.e.,
  17 $$ \begin{aligned} D_{\mathrm{w}}^{\text{conv}} =& D_{\text{tis}}^{\text{MC}}\; \left (\bar \mu_{\text{en}}/\rho \right)_{\text{w,tis}}\;\frac{\Psi_{\mathrm{w}}}{\Psi_{\text{tis}}}\\ k_{\Psi} =& \frac{\Psi_{\mathrm{w}}}{\Psi_{\text{tis}}}  \end{aligned}  $$

## Conclusions

The use of the Monte Carlo method for calculations in radiotherapy dosimetry has become the most efficient and consistent tool for simulations in most of the fields related to the speciality, from basic dosimetric quantities, like stopping-power ratios and perturbation correction factors for reference ionization chamber dosimetry, to fully realistic simulations of clinical accelerators, detectors and patient treatment planning. Its accurate use requires consistency in the data throughout the entire dosimetry chain, and the recent updates of key dosimetric data by ICRU Report 90 are necessary in reference dosimetry. Although data consistency is probably less critical for treatment planning, their implementation also in this field is advised. There are, however, a number of other issues raised throughout this work to conclude with the recommendation that no MC calculation should be considered free of errors. This is particularly important with regard to applications in MC treatment planning, where the uncertainties involved still remain “uncertain”, a general problem that is also applicable to other methods and algorithms used in different types of treatment planning systems.

## Footnotes

1 Recall that, strictly speaking, the quantity absorbed dose cannot be *measured*; it is always *determined* from measurements of related quantities, like charge, current, heat, chemical changes, etc., using appropriate correction and conversion factors.

2 It should be noted that ICRU Report 85 [4] included an incomplete definition of the quantity restricted cema, which did not consider the track-end term; this definition was, however, updated in ICRU Report 90 [5].

3 A description of *PENELOPE*, *EGSnrc* and other general MC systems like FLUKA, GEANT, MCNP, etc, as well as their applications in radiation dosimetry in the fields of radiotherapy, nuclear medicine and radiodiagnostics can be found in Chapter 8 of ref. [3].
